# Bifurcation Analysis of a Micro-Machined Gyroscope with Nonlinear Stiffness and Electrostatic Forces

**DOI:** 10.3390/mi12020107

**Published:** 2021-01-22

**Authors:** Huabiao Zhang, Xinye Li, Lijuan Zhang

**Affiliations:** 1 School of Mechanical Engineering, Tianjin University of Commerce, Tianjin 300134, China; 2 School of Mechanical Engineering, Hebei University of Technology, Tianjin 300401, China; 1994109@hebut.edu.cn; 3 School of Automobile and Transportation, Tianjin University of Technology and Education, Tianjin 300222, China; 2020050004@tute.edu.cn

**Keywords:** micro-machined gyroscope, nonlinear dynamics, static pull-in analysis, singularity analysis, bifurcation of periodic solutions

## Abstract

The bifurcation of the periodic response of a micro-machined gyroscope with cubic supporting stiffness and fractional electrostatic forces is investigated. The pull-in phenomenon is analyzed to show that the system can have a stable periodic response when the detecting voltage is kept within a certain range. The method of averaging and the residue theorem are employed to give the averaging equations for the case of primary resonance and 1:1 internal resonance. Transition sets on the driving/detecting voltage plane that divide the parameter plane into 12 persistent regions and the corresponding bifurcation diagrams are obtained via the singularity theory. The results show that multiple solutions of the resonance curves appear with a large driving voltage and a small detecting voltage, which may lead to an uncertain output of the gyroscope. The effects of driving and detecting voltages on mechanical sensitivity and nonlinearity are analyzed for three persistent regions considering the operation requirements of the micro-machined gyroscope. The results indicate that in the region with a small driving voltage, the mechanical sensitivity is much smaller. In the other two regions, the variations in the mechanical sensitivity and nonlinearity are analogous. It is possible that the system has a maximum mechanical sensitivity and minimum nonlinearity for an appropriate range of detecting voltages.

## 1. Introduction

Micro-machined gyroscopes are widely used because of their small size, light weight, simple structure, low manufacturing cost, and high reliability [1]. The performance of micro-machined gyroscopes is closely related to their dynamical characteristics. The existence of nonlinear factors makes their dynamical response more complex, so the nonlinear dynamics of micro-machined gyroscopes have attracted the attention of researchers over the last decades.

Braghin et al. [2] investigated a lumped parameter model of a MEMS gyroscope by using semi-analytical and numerical methods. The existence of hardening nonlinear stiffness was confirmed through experiments. Martynenko et al. [3] studied a MEMS gyroscope with a ring resonator supported by a flexible torsion system by considering the material nonlinearity. The results showed that the nonlinear elastic properties led to additional errors, unstable branches of resonance curves, and quenching. Ref. [4] also analyzed the nonlinear vibrations of an MEMS tuning fork gyroscope with an elastic rod. The influence of the slowly changing angular velocity was taken into consideration. Li et al. [5,6] considered the nonlinear dynamics of a machined gyroscope with nonlinear stiffness and delayed feedback control and analyzed the effects of system parameters and feedback gains. Mojahedi et al. [7] studied the static and dynamical characteristics of micro-/nano-mechanical gyroscopes with nonlinear electrostatic forces and intermolecular forces. The effects of parameters on static/dynamic deflection, natural frequency, and instability were discussed numerically. Nitzan et al. [8] discussed the influence of cubic nonlinearity on the error of rate-integrated gyroscopes. Hamed et al. [9] considered an MEMS gyroscope with linear and nonlinear parametric excitations. Considering the parametric resonance and 1:1 internal resonance, the steady-state response and its stability were discussed. With the existence of parameter uncertainties and noise measurement, comparisons between optimal linear feedback control and active control via negative nonlinear cubic velocity feedback were given. Shang et al. [10,11] considered a machined gyroscope with parametric excitation and cubic nonlinearity. The Hopf bifurcation condition was obtained, and a numerical simulation was used to study the driving and detecting amplitudes. Awrejcewicz et al. [12] studied a micromechanical gyroscope with torsion joints, considering the effects of stiffness and joint nonlinearity, and performed a multi-scale analysis of the steady-state response of the system in the case of primary and internal resonance.

Electrostatic force is also an important nonlinearity in these systems. Micro-machined gyroscopes are usually driven and detected by capacitors. The nonlinear effect of the electrostatic force of the driving and detecting capacitors cannot be negligible. Considering the nonlinearity of electrostatic forces, Lestev et al. [13] studied the steady-state response of a micro-machined gyroscope. Tsai et al. [14] investigated a micro-machined gyroscope with nonlinear stiffness and electrostatic forces, and showed the unstable region on the driving/detecting frequency plane. Then, the periodic solution and chaos were analyzed. Kacem et al. [15] established a continuum model of a resonant gyroscope. The steady-state response of the system was studied based on the Galerkin method and the perturbation method. Lajimi et al. [16] studied a micro-machined gyroscope consisting of a beam and a rigid body, and solved the equations with a multi-scale method. The response was analyzed for both amplitude modulation and frequency modulation. Ref. [17] also investigated the mechanical–thermal noise of a micro-machined gyroscope. Sharma et al. [18] numerically and experimentally analyzed the pull-in phenomenon of a micro-machined gyroscope and discussed the effects of the dynamic pull-in voltage and the measured angular velocity. Tehrani et al. [19] experimentally described the performance of an MEMS gyroscope with different forms of mechanical and electrostatic nonlinearities, and found that a low-angle random walk can be achieved even though the gyroscope’s drive mode exhibits high amplitude–frequency dependence, and that the bias instability is largely independent of the operating regime. Ouakad [20] investigated the influences of the size effects on the pull-in phenomena and the primary resonance response of an MEMS gyroscope model given by the modified coupled-stress non-classical mechanics theory.

A micro-machined gyroscope system is a typical nonlinear vibration system with 1:1 internal resonance [8,10,12]. This 1:1 internal resonance is one of the important research directions of nonlinear dynamics [21,22]. It occurs in numerous mechanical systems, such as cables [23], beams [24,25], circular or square plates [26,27], etc. Many investigations, including those of periodic response [23], backbone curves [21,22], bifurcation characteristics [28], and chaos [29], have been done on this subject. The singularity theory is an effective method for bifurcation investigation. Based on this theory, the transition sets on the unfolding parameter plane and the corresponding bifurcation diagrams with different topological structures can be given [30]. Chen and Langford [31] studied a generally non-linear Mathieu equation and obtained six typical bifurcating response curves by setting the system parameters as the unfolding parameters. By using the singularity theory, Qin et al. [23] studied the bifurcation of an elastic cable with 1:1 internal resonance. Hou et al. [32] investigated the bifurcation modes of a periodic solution in a duffing system under constant force and harmonic excitation. Li et al. [33] considered the bifurcation of a coupled pitch–roll ship model with quadratic and cubic nonlinearity.

Generally speaking, the electrostatic force of a micro-machined gyroscope in the detecting direction is a fraction of the displacement; it has conventionally been developed into a Taylor series of the displacement in recent studies. However, Taylor expansion is accurate only in the neighborhood of the expansion point. For micro-machined gyroscopes, the amplitude of the detecting direction tends to be close to the gap of the capacitance to improve its sensitivity. In this case, the conclusions must be inaccurate with Taylor expansion. Moreover, most of the existing studies have discussed the influence of parameters on the response. Few studies have focused on the singularity analysis of micro-machined gyroscope systems.

In this paper, we propose an investigation of the bifurcation of a micro-machined gyroscope with nonlinear stiffness and electrostatic forces. In Section 2, the Lagrange equation is used to establish the dynamical equations of the micro-machined gyroscope. The pull-in phenomenon in the detecting direction is analyzed in Section 3. In Section 4, the method of averaging is employed to obtain the analytical solutions without Taylor expansion. Then, the influences of the driving and detecting voltages on the bifurcation characteristics of the amplitude–frequency response are discussed with the singularity theory in Section 5. Finally, the conclusion summarizes the work. Our innovation is the singularity analysis of a micro-machined gyroscope system with consideration of the fractional electrostatic force without Taylor expansion.

## 2. Physical Model of the Micro-Machined Gyroscope and Its Mathematical Description

**Figure 1:**
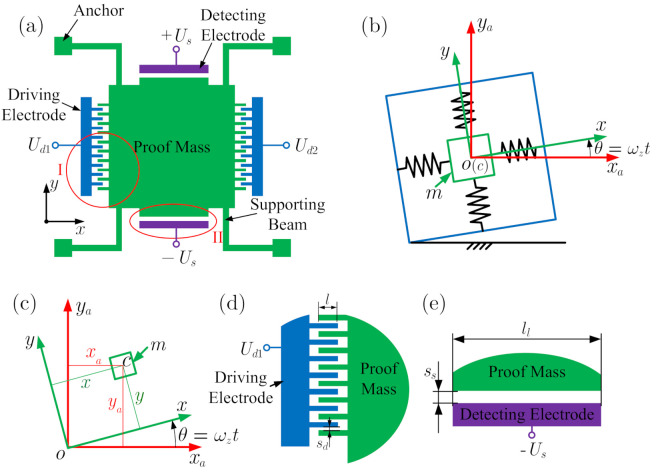
Model of the micro-machined gyroscope system. (**a**) The schematic representation of the micro-machined gyroscope. (**b**) Model of the gyroscope as a spring-mass system. (**c**) The relations between the displacements of the driving/detecting coordinate system and the inertial coordinate system. (**d**) The enlargement of I in Figure 1a: the driving structure. (**e**) The enlargement of II in Figure 1a: the detecting structure.

Figure 1 gives a model of a micro-machined gyroscope. Figure 1a shows the schematic representation of the micro-gyroscope, and Figure 1b shows the equivalent spring-mass model. In Figure 1b, *m* is the proof mass of the sensitive structure. $x_{a}-o-y_{a}$ is the inertial coordinate system, and x−o−y is the driving/detecting coordinate system of the micro-machined gyroscope. The origin *o* is located at the center of the proof mass, as the system is in static balance, as shown in Figure 1b. *x* is the driving direction, *y* is the detecting direction, and *z* is the direction of vertical paper facing outwards. θ is the angular displacement of the gyroscope around the *z*-axis, and $\omega_{z}$ is the angular velocity being measured. As mentioned in Ref. [34], the angular velocity $\omega_{z}$ is assumed to be constant. Therefore, Euler’s second law of motion can be omitted in the considerations. Based on the relations between the displacements of the driving/detecting coordinate system and the inertial coordinate system shown in Figure 1c, the displacements of the proof mass in the inertial coordinate system can be given as

(1) $$\begin{array}{c} x_{a}=x\cos\omega_{z}t-y\sin\omega_{z}t,\\ y_{a}=x\sin\omega_{z}t+y\cos\omega_{z}t. \end{array}$$

The velocity of the proof mass can be obtained from the derivative of Equation (1) with respect to time as

(2) $$\begin{array}{c} {\dot{x}}_{a}=\left(\dot{x}-y\omega_{z}\right)\cos\omega_{z}t+\left(x\omega_{z}+\dot{y}\right)\sin\omega_{z}t,\\ {\dot{y}}_{a}=\left(x\omega_{z}+\dot{y}\right)\cos\omega_{z}t+\left(\dot{x}-y\omega_{z}\right)\sin\omega_{z}t. \end{array}$$

Then, the kinetic energy of the proof mass is

(3) $$T=\frac{1}{2}m\left({\dot{x}}_{a}^{2}+{\dot{y}}_{a}^{2}\right).$$

The supporting beams of the micro-machined gyroscope have cubic nonlinear stiffness, and the elastic potential energy can be obtained as

(4) $$V=\frac{1}{2}k_{x}x^{2}+\frac{1}{2}k_{y}y^{2}+\frac{1}{4}\alpha_{x}x^{4}+\frac{1}{4}\alpha_{y}y^{4},$$

where $k_{x},k_{y},\alpha_{x},\alpha_{y}$ are the linear and nonlinear stiffness coefficients in the driving and detecting directions, respectively. The Rayleigh dissipation function of the system is

(5) $$D=\frac{1}{2}c_{x}{\dot{x}}^{2}+\frac{1}{2}c_{y}{\dot{y}}^{2},$$

where $c_{x},c_{y}$ are the damping coefficients in the driving and detecting directions, respectively. According to the Lagrange equation, the motion equations of the system are given as

(6) $$\begin{array}{c} m\ddot{x}-2m\omega_{z}\dot{y}+\left(k_{x}-m{\omega_{z}}^{2}\right)x+\alpha_{x}x^{3}+c_{x}\dot{x}=F_{x},\\ m\ddot{y}+2m\omega_{z}\dot{x}+\left(k_{y}-m{\omega_{z}}^{2}\right)y+\alpha_{y}y^{3}+c_{y}\dot{y}=F_{y}, \end{array}$$

where $-2m\omega_{z}\dot{y}$ and $2m\omega_{z}\dot{x}$ are the Coriolis forces, which are also known as the gyro forces of the gyroscope. $F_{x}$ and $F_{y}$ denote the electrostatic forces of the driving and detecting directions, respectively. The driving and detecting structures are shown in Figure 1d,e, respectively. When the proof mass has a displacement *x*, the two capacitances in the driving direction are

(7) $$C_{d1}=\frac{2n\eta a(l+x)}{s_{d}},\;\;\;\;\;\;\;\;\;C_{d2}=\frac{2n\eta a(l-x)}{s_{d}},$$

where *n* is the number of combs, *l* is the overlap length of the driving combs, *a* is the thickness of the driving comb, and $s_{d}$ is the clearance between the combs. η is the dielectric constant. The driving voltages are

(8) $$U_{d1}=U_{d0}+U_{da}\cos\omega t,U_{d2}=U_{d0}-U_{da}\cos\omega t.$$

Then, the electrostatic force of the driving direction can be given as

(9) $$F_{x}=\frac{1}{2}{U_{d1}}^{2}\frac{\partial C_{d1}}{\partial x}+\frac{1}{2}{U_{d2}}^{2}\frac{\partial C_{d2}}{\partial x}=\frac{4n\eta \;aU_{d0}\;U_{da}\;\cos\omega \;t}{s_{d}}.$$

Similarly, when the proof mass has a displacement *y*, the two capacitances in the detecting direction are

(10) $$C_{s1}=\frac{\eta l_{l}l_{w}}{s_{s}+y},C_{s2}=\frac{\eta l_{l}l_{w}}{s_{s}-y},$$

where $l_{l}$ and $l_{w}$ are the length and thickness of the detecting electrode. $s_{s}$ is the capacitance gap. Then, the electrostatic force of the detecting direction can be given as

(11) $$F_{y}=\frac{1}{2}{U_{s}}^{2}\frac{\partial C_{s1}}{\partial x}+\frac{1}{2}{U_{s}}^{2}\frac{\partial C_{s2}}{\partial x}=\frac{2\eta l_{l}l_{w}s_{s}U_{s}^{2}y}{{\left(s_{s}-y\right)}^{2}{\left(s_{s}+y\right)}^{2}}.$$

In the stiffness term of Equation (6), $m{\omega_{z}}^{2}$ can be ignored, as $k_{x}\gg m{\omega_{z}}^{2},k_{y}\gg m{\omega_{z}}^{2}$. By setting $X=x/s_{s},Y=y/s_{s},\tau =\omega_{0}t$, the motion equations can be rewritten in the non-dimensional form as

(12) $$\begin{array}{c} X^{\prime \prime }-\varepsilon \Omega_{z}Y^{\prime }+{\omega_{x}}^{2}X+\beta_{X}X^{3}+\varepsilon \xi_{X}X^{\prime }=e_{X}\cos\Omega \tau ,\\ Y^{\prime \prime }+\varepsilon \Omega_{z}X^{\prime }+Y+\beta_{Y}Y^{3}+\varepsilon \xi_{Y}Y^{\prime }=\frac{e_{Y}Y}{{\left(1-Y\right)}^{2}{\left(1+Y\right)}^{2}}, \end{array}$$

where

$$\begin{array}{c} \varepsilon \Omega_{z}=\frac{2\omega_{z}}{\omega_{0}},{\omega_{x}}^{2}=\frac{k_{x}}{m{\omega_{0}}^{2}},{\omega_{0}}^{2}=\frac{k_{y}}{m},\beta_{X}=\frac{{s_{s}}^{2}\alpha_{x}}{m{\omega_{0}}^{2}},\beta_{Y}=\frac{{s_{s}}^{2}\alpha_{y}}{m{\omega_{0}}^{2}},\\ \varepsilon \xi_{X}=\frac{c_{x}}{m\omega_{0}},\varepsilon \xi_{Y}=\frac{c_{y}}{m\omega_{0}},e_{X}=\frac{4\eta naU_{d0}U_{da}}{m{\omega_{0}}^{2}s_{s}s_{d}},e_{Y}=\frac{2\eta l_{l}l_{w}s_{s}U_{s}^{2}}{m{\omega_{0}}^{2}{s_{s}}^{4}}, \end{array}$$

where 0<ε≪1 is a small parameter, and $e_{X},e_{Y}$ are the non-dimensional parameters relevant to the driving and detecting voltages, respectively. Moreover, since $s_{s}$ is a certain value, Equation (12) has a constraint Y≤1.

## 3. Pull-In Analysis of the Detecting Equation

It is known that the pull-in phenomenon, which means that stable equilibriums of the system cease to exist, takes place when the detecting voltage is greater than a critical value. The proof mass is contacted with the detecting electrode, and the system cannot work. Therefore, we focus on pull-in analysis of the motion equation of the detecting direction in this section. By ignoring items with ε and setting $Y=Y_{1},Y^{\prime }=Y_{2}$, the motion equation in the detecting direction can be written in the form of state equations as

(13) $$\begin{array}{c} {Y_{1}}^{\prime }=Y_{2},\\ {Y_{2}}^{\prime }=-Y_{1}-\beta_{Y}{Y_{1}}^{3}+\frac{e_{Y}Y_{1}}{{\left(1-Y_{1}\right)}^{2}{\left(1+Y_{1}\right)}^{2}}. \end{array}$$

Obviously, Equation (13) is a Hamiltonian system, and the equilibriums of the equation are determined by Equation (14).

(14) $$\begin{array}{c} -Y_{1}-\beta_{Y}{Y_{1}}^{3}+\frac{e_{Y}Y_{1}}{{\left(1-Y_{1}\right)}^{2}{\left(1+Y_{1}\right)}^{2}}=0,\\ Y_{2}=0. \end{array}$$

**Figure 2:**
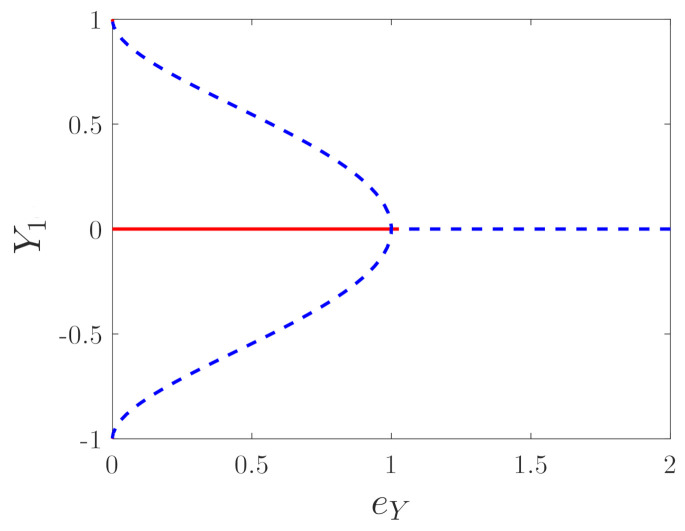
Bifurcation diagram of the equilibrium point with variation of $e_{Y}$, $\beta_{Y}=0.05$.

Figure 2 shows the bifurcation diagram of the equilibriums. The fork bifurcation occurs with the change in $e_{Y}$. $e_{Y}=1$ is the bifurcation point. As $e_{Y}>1$, the system has an unstable equilibrium (0,0), and as $e_{Y}<1$, the system has three equilibriums $P_{0}\left(0,0\right),P_{1}\left(Y_{s},0\right),P_{2}\left(-Y_{s},0\right)$, where

(15) $$\begin{array}{c} Y_{s}={\left(\frac{2\;\beta_{Y}-1}{3\beta_{Y}}-\;\frac{1}{3}\sqrt{1+2\;{\beta_{Y}}^{-1}+{\beta_{Y}}^{-2}}\left(\cos\frac{\alpha }{3}-\sqrt{3}\sin\frac{\alpha }{3}\right)\right)}^{1/2},\\ \cos\alpha =-\;\frac{\left(2\;{\beta_{Y}}^{3}-27e_{Y}\;{\beta_{Y}}^{2}+6\;{\beta_{Y}}^{2}+6\;\beta_{Y}+2\right)}{2{\left(\beta_{Y}+1\right)}^{3}}. \end{array}$$

Here, $P_{0}$ is the center, and the other two equilibriums $P_{1},P_{2}$ are saddle points. In order to deeply understand the dynamics of Equation (13), the potential energy function and the Hamiltonian function can be obtained as

(16) $$V=\frac{1}{2}\;{Y_{1}}^{2}+\frac{1}{4}\;\beta_{Y}\;{Y_{1}}^{4}-\frac{1}{4}e_{Y}\;\left(\frac{1}{1-Y_{1}}+\frac{1}{1+Y_{1}}\right),$$

(17) $$H=\frac{1}{2}\;{Y_{2}}^{2}+\frac{1}{2}\;{Y_{1}}^{2}+\frac{1}{4}\;\beta_{Y}\;{Y_{1}}^{4}-\frac{1}{4}e_{Y}\;\left(\frac{1}{1-Y_{1}}+\frac{1}{1+Y_{1}}\right).$$

**Figure 3:**
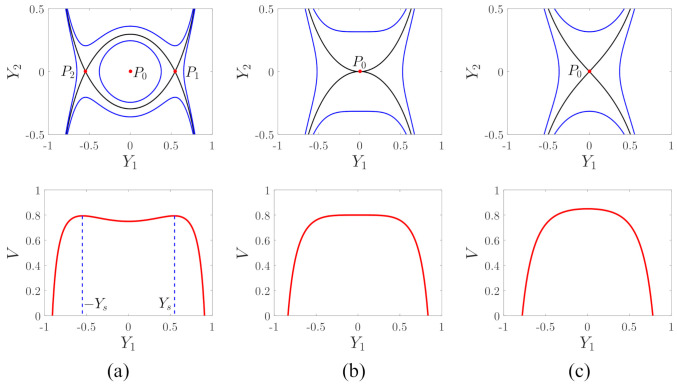
Phase trajectories and potential energy curves of the system; (**a**) $e_{Y}=0.5$, (**b**) $e_{Y}=1$, and (**c**) $e_{Y}=1.5$.

Figure 3 shows the phase trajectories and potential energy curves of the system. As $e_{Y}\geq 1$, the response of the undisturbed system will tend to infinity no matter what the initial value is. For the case of $e_{Y}<1$, the phase trajectory of the undisturbed system is a closed curve, which means that the system has a periodic response if the initial value is between two heteroclinic trajectories in the phase plane. When the initial value is given outside the two heteroclinic trajectories, the response of the system will also tend to infinity. The area between the two heteroclinic trajectories is the security region of the undisturbed system. As $e_{Y}\geq 1$, the potential energy curve has only a maximum point, and it is obviously impossible to have stable motion. As $e_{Y}<1$, the potential energy curve has a potential that is close to $Y_{1}=0$. If the amplitude of the system response is less than $Y_{s}$, the motion is periodic.

**Figure 4:**
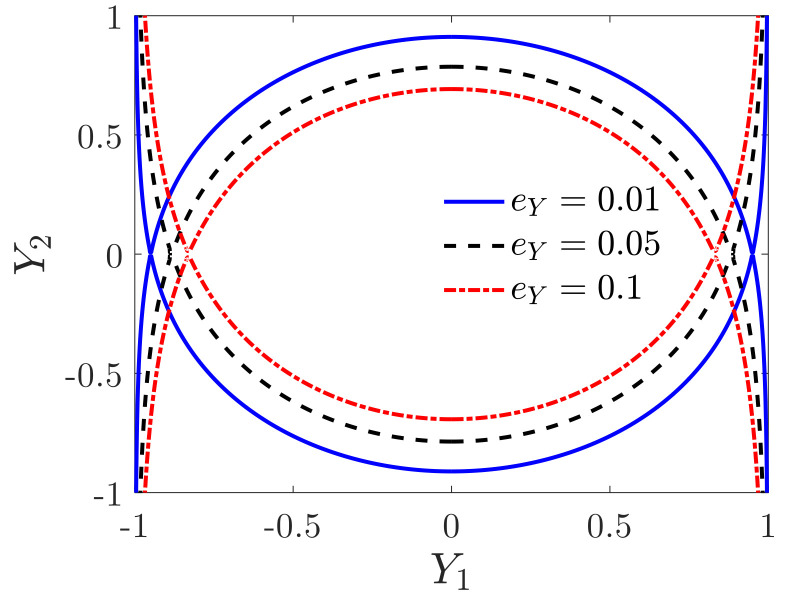
The effects of the detecting voltage on the security region of the undisturbed system.

Since the system contains a constraint $Y_{1}\leq 1$, if the response of the system is unstable, finally, $Y_{1}=1$, the capacitance gap in the detecting direction is zero, and the proof mass and the detecting electrode are closely attached, which is commonly known as pull-in. Figure 4 shows the influences of the detecting voltage on the security region of the undisturbed system. The area between the two heteroclinic trajectories is the security region. It can be seen that the security region of the undisturbed system decreases with the detecting voltage.

## 4. Approximate Analytical Solution of the Micro-Machined Gyroscope System

The performance of micro-machined gyroscope is closely related to its periodic response. In this section, the solutions are analytically solved with the method of averaging. By setting $e_{X}=\varepsilon E_{X},\;\;\;\;\;e_{Y}=\varepsilon E_{Y}$, $\beta_{X}=\varepsilon \gamma_{X},\beta_{Y}=\varepsilon \gamma_{Y}$, Equation (12) can be rewritten as

(18) $$\begin{array}{c} X^{\prime \prime }-\varepsilon \Omega_{z}Y^{\prime }+{\omega_{x}}^{2}X+\varepsilon \gamma_{X}X^{3}+\varepsilon \xi_{X}X^{\prime }=\varepsilon E_{X}\cos\Omega \tau ,\\ Y^{\prime \prime }+\varepsilon \Omega_{z}X^{\prime }+Y+\varepsilon \gamma_{Y}Y^{3}+\varepsilon \xi_{Y}Y^{\prime }=\frac{\varepsilon E_{Y}Y}{{\left(1-Y\right)}^{2}{\left(1+Y\right)}^{2}}. \end{array}$$

The solutions of Equation (18) are assumed as [35]

(19) $$\begin{array}{c} X=A_{1}\cos\phi_{1}=A_{1}\cos\left(\Omega \tau +\theta_{1}\right),\\ Y=A_{2}\cos\phi_{2}=A_{2}\cos\left(\Omega \tau +\theta_{2}\right), \end{array}$$

where $A_{1}$ and $A_{2}$ are the amplitudes, and $\theta_{1}$ and $\theta_{2}$ are phase angles in the *x* and *y* directions, respectively, which can be determined by the following averaging equations:

(20) $$\begin{array}{c} {A^{\prime }}_{1}=-\frac{1}{2\pi \Omega }\int_{-\pi }^{\pi }f_{1}\sin\phi_{1}d\phi_{1},\\ A_{1}{\theta^{\prime }}_{1}=-\frac{1}{2\pi \Omega }\int_{-\pi }^{\pi }f_{1}\cos\phi_{1}d\phi_{1},\\ {A^{\prime }}_{2}=-\frac{1}{2\pi \Omega }\int_{-\pi }^{\pi }\left(f_{21}-f_{22}\right)\sin\phi_{2}d\phi_{2},\\ A_{2}{\theta^{\prime }}_{2}=-\frac{1}{2\pi \Omega }\int_{-\pi }^{\pi }\left(f_{21}-f_{22}\right)\cos\phi_{2}d\phi_{2}, \end{array}$$

where

$$\begin{array}{c} f_{1}=\varepsilon \Omega_{z}Y^{\prime }-\varepsilon \gamma_{X}X^{3}-\varepsilon \xi_{X}X^{\prime }+\varepsilon E_{X}\cos\Omega \tau +\left(\sigma_{1}+\sigma_{2}\right)X,\\ f_{21}=-\varepsilon \Omega_{z}X^{\prime }-\varepsilon \gamma_{Y}Y^{3}-\varepsilon \xi_{Y}Y^{\prime }+\sigma_{2}Y,\\ f_{22}=\frac{\varepsilon E_{Y}Y}{{\left(1-Y\right)}^{2}{\left(1+Y\right)}^{2}}, \end{array}$$

where $\sigma_{1}$ and $\sigma_{2}$ are the two detuning parameters that describe the nearness of primary resonance and 1:1 internal resonance:

$$\Omega^{2}=1+\varepsilon \sigma_{1},{\omega_{x}}^{2}=1-\varepsilon \sigma_{2}$$

Note that $f_{1}$ and $f_{21}$ are polynomials, and $f_{22}$ is a fraction. The integration of Equation (20) is easy to solve, except for the fractional part. Thus, we set

(21) $$\begin{array}{c} {\bar{f}}_{A}=\frac{1}{2\pi \Omega }\int_{-\pi }^{\pi }f_{22}\sin\phi_{2}d\phi_{2},\\ f_{A}=\frac{1}{2\pi \Omega }\int_{-\pi }^{\pi }f_{22}\cos\phi_{2}d\phi_{2}. \end{array}$$

The integrand of ${\bar{f}}_{A}$ is

(22) $$f_{22}\sin\phi_{2}\;=\;\frac{\varepsilon E_{Y}A_{2}\cos\phi_{2}\sin\phi_{2}}{{\left(1-A_{2}\cos\phi_{2}\right)}^{2}{\left(1+A_{2}\cos\phi_{2}\right)}^{2}}.$$

It is obvious that ${\bar{f}}_{A}=0$, since Equation (22) is an odd function about $\phi_{2}$ and the integral interval [−π,π] is symmetric about zero. On the calculation of $f_{A}$, by setting $z=\exp(i\phi_{2})$, one has

(23) $$\cos\phi_{2}=\;\frac{z^{2}+1}{2z},\sin\phi_{2}=\frac{z^{2}-1}{2iz}.$$

According to the residue theorem [36], substituting Equation (23) into $f_{A}$ gives

(24) $$\begin{array}{c} f_{A}=\frac{1}{2\pi \Omega }\int_{-\pi }^{\pi }f_{22}\cos\phi_{2}d\phi_{2}=\frac{1}{2\pi \Omega }\int_{0}^{2\pi }f_{22}\cos\phi_{2}d\phi_{2}\\ =\frac{1}{2\pi \Omega }\oint_{|z|=1}f(z)dz=\frac{i}{\Omega }\sum \mathrm{Res}[f\left(z\right),z_{k}], \end{array}$$

where

(25) $$f\left(z\right)=\frac{-4\;iE_{Y}\;A_{2}z{\left(z^{2}+1\right)}^{2}}{{\left(z^{2}A_{2}-2\;z+A_{2}\right)}^{2}{\left(z^{2}A_{2}+2\;z+A_{2}\right)}^{2}},$$

where $z_{k}$ are the isolated singularities of f(z) within the unit circle. Due to $A_{2}\leq 1$, Equation (25) has two secondary poles in the unit circle, which are

(26) $$z_{k}=\pm \frac{\sqrt{2-{A_{2}}^{2}-2\;\sqrt{1-{A_{2}}^{2}}}}{A_{2}}.$$

Substituting Equation (26) into Equation (24) leads to

(27) $$f_{A}=\frac{E_{Y}A_{2}\sqrt{1-{A_{2}}^{2}}\;}{2\Omega {\left(1-{A_{2}}^{2}\right)}^{2}}.$$

Then, the averaging equations are obtained as

(28) $$\begin{array}{c} A^{\prime }=\;\frac{\Omega_{z}\Omega \;A_{2}\cos\left(\theta_{1}-\theta_{2}\right)-\Omega \;A_{1}\xi_{X}-E_{X}\sin\theta_{1}}{2\Omega },\\ A_{1}{\theta_{1}}^{\prime }=\;\frac{-4\;\Omega_{z}\Omega \;A_{2}\sin\left(\theta_{1}-\theta_{2}\right)+3\;\gamma_{X}{A_{1}}^{3}-4\;E_{X}\cos\theta_{1}-4\;A_{1}\left(\sigma_{1}+\sigma_{2}\right)}{8\Omega },\\ {A_{2}}^{\prime }=\frac{-\;\Omega_{z}A_{1}\Omega \;\cos\left(\theta_{1}-\theta_{2}\right)-\Omega \;A_{2}\xi_{Y}}{2\Omega },\\ A_{2}{\theta_{2}}^{\prime }=\frac{-4\;\Omega_{z}A_{1}\Omega \;\sin\left(\theta_{1}-\theta_{2}\right)+3\;\gamma_{Y}{A_{2}}^{3}-4\;A_{2}\sigma_{1}}{8\Omega }-f_{A}. \end{array}$$

By setting $A_{1}^{\prime }=A_{2}^{\prime }=\theta_{1}^{\prime }=\theta_{2}^{\prime }=0$ and eliminating the trigonometric function terms in Equation (28), the bifurcation equations for the amplitude $A_{1},A_{2}$ can be given as

(29) $$\begin{array}{c} {\left(3\;{A_{2}}^{4}\gamma_{Y}-3\;{A_{1}}^{4}\gamma_{X}+8\;\Omega \;f_{A}A_{2}+4\;{A_{1}}^{2}\sigma_{1}+4\;{A_{1}}^{2}\sigma_{2}-4\;{A_{2}}^{2}\sigma_{1}\right)}^{2}\\ +16\Omega^{2}{\left({A_{1}}^{2}\xi_{X}+{A_{2}}^{2}\xi_{Y}\right)}^{2}-16{A_{1}}^{2}{E_{X}}^{2}=0, \end{array}$$

(30) $$16\Omega^{2}{A_{2}}^{2}{\xi_{Y}}^{2}+(3\;\gamma_{Y}{A_{2}}^{3}+8\;f_{A}\;\Omega -4\;A_{2}\sigma_{1}{)}^{2}-16G^{2}\Omega^{2}{A_{1}}^{2}=0.$$

To study the stability of the periodic response, we set $A_{1}=A_{10}+p_{1},\theta_{1}=\theta_{10}+p_{2},A_{2}=A_{20}+p_{3},\theta_{2}=\theta_{20}+p_{4}$, where $A_{10},A_{20},\theta_{10},\theta_{20}$ are the steady-state solutions and $p_{i}\left(i=1,2,3,4\right)$ are small perturbations. Substituting them into Equation (28), and ignoring the high-order terms of $p_{i}$, one has

(31) $$p^{\prime }=\mathrm{Mp},$$

where $p={\left[p_{1},p_{2},p_{3},p_{4}\right]}^{T}$, and M is the Jacobian matrix of the periodic response. The expressions of its elements are given in the  Appendix A. The stability of the steady-state solutions can be determined by the eigenvalues of M. The steady solution is stable if and only if the real part of all eigenvalues is negative. A positive real part of the eigenvalue leads to unstable solutions. The steady solution loses its stability as one real eigenvalue changes its sign, and the saddle–node bifurcation occurs. A pair of complex conjugate eigenvalues passing the imaginary axis of the complex plane at the bifurcation point leads to Hopf bifurcation [37].

**Figure 5:**
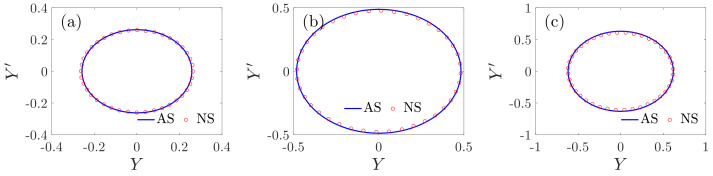
Comparisons between the analytical solutions and the numerical solutions; (**a**) $E_{X}=10$, $E_{Y}=30$, and $\sigma_{1}=-30$, (**b**) $E_{X}=25$, $E_{Y}=30,$ and $\sigma_{1}=-20$, and (**c**) $E_{X}=40$, $E_{Y}=30$, and $\sigma_{1}=-40$. AS: analytical solutions, NS: numerical solutions.

**Figure 6:**
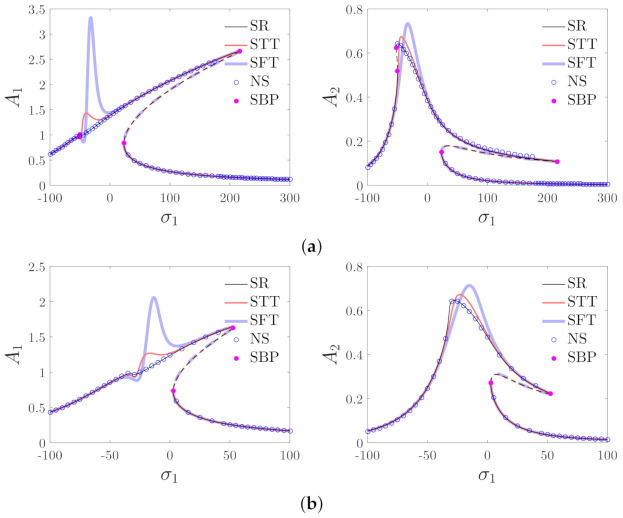
Comparisons between the solutions based on the residue theorem and Taylor expansion; (**a**) $E_{X}=40$ and $E_{Y}=30$ and (**b**) $E_{X}=25$, and $E_{Y}=20$. SR: solutions based on the residue theorem, STT: solutions based on the third-order Taylor expansion, SFT: solutions based on the fifth-order Taylor expansion, NS: numerical solutions, SBP: the saddle-node bifurcation point.

If there are no special instructions, the values of the parameters in this paper are chosen as $\xi_{X}=\xi_{Y}=15,\gamma_{X}=\gamma_{Y}=50,\Omega_{z}=10,\sigma_{2}=50,\varepsilon =0.001$. Figure 5 shows the comparisons between the analytical solutions and the numerical solutions. The numerical solutions are given by the fourth-order Runge–Kutta method. It can be observed that the analytical solutions are in good agreement with the numerical solutions. Based on Equations (29) and (30) and the stability analysis, Figure 6 provides the results based on both the residue theorem and Taylor expansion. It is shown that the results obtained by the residue theorem are more accurate than the results based on Taylor expansion.

**Figure 7:**
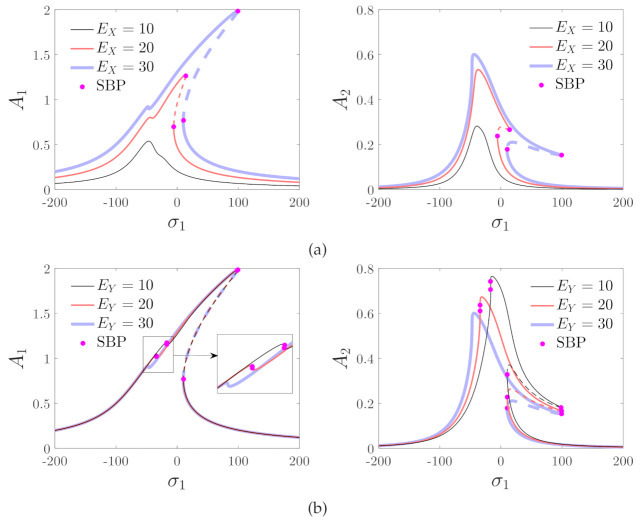
Effects of driving and detecting voltages on the response of the micro-machined gyroscope. (**a**) The effects of driving voltage $\left(E_{Y}=30\right)$. (**b**) The effects of detecting voltage $\left(E_{X}=30\right)$. SBP denotes the saddle–node bifurcation point.

The resonance curves with different values of the driving and detecting voltages are shown in Figure 7. The figure shows that the topological structures of the curves are greatly different when $E_{X},E_{Y}$ are varied. Therefore, it is necessary to analyze the bifurcation characteristics to illustrate the effects of the driving and detecting voltages on the resonance curves.

## 5. Bifurcation Analyses

It can be obtained from the Equation (30) that

$${A_{1}}^{2}=\frac{16\Omega^{2}{A_{2}}^{2}{\xi_{Y}}^{2}+(3\;\beta_{Y}{A_{2}}^{3}+8\;f_{A}\;\Omega -4\;A_{2}\sigma_{1}{)}^{2}}{16G^{2}\Omega^{2}}=A_{12}.$$

Substituting it into Equation (29) gives

(32) $$\begin{array}{c} \;f\left(A_{2},\sigma_{1}\right)={\left(3\;{A_{2}}^{4}\beta_{Y}-3\;{A_{12}}^{2}\beta_{X}+8\;\Omega \;f_{A}A_{2}+4\;A_{12}\sigma_{1}+4\;A_{12}\sigma_{2}-4\;{A_{2}}^{2}\sigma_{1}\right)}^{2}\\ +16\Omega^{2}{\left(A_{12}\xi_{X}+{A_{2}}^{2}\xi_{Y}\right)}^{2}-16A_{12}{E_{X}}^{2} \end{array}.$$

The above equation is an algebraic equation about $A_{2}$. Its solutions may bifurcate when parameters such as $\sigma_{1}$ and $\sigma_{2}$ are varied, since it is nonlinear. In this section, we have conducted a bifurcation analysis of Equation (32). For simplicity, $\sigma_{1}$ in Equation (32) is regarded as the bifurcation parameter, and $\sigma_{2}$ is fixed in the following discussion.

Then, the singularity theory [30,38] is employed to analyze the effects of $E_{X}$ and $E_{Y}$ on the bifurcation characteristics of Equation (32). The transition sets of Equation (32) are defined as D=B∪H∪DL, where B,H,DL denote the bifurcation sets, the hysteresis sets, and the double limit point sets, respectively. Their expressions are given in Table 1.

**Figure 8:**
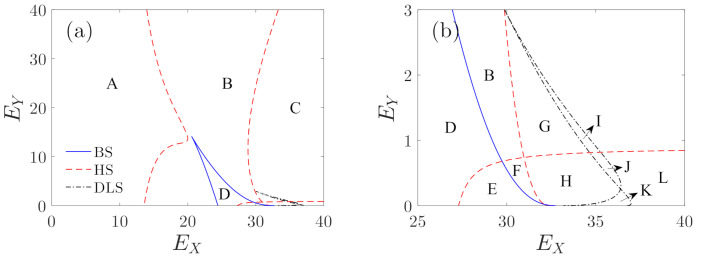
(**a**) Transition sets on the $E_{X}-E_{Y}$ plane. (**b**) The partial enlargement of subplot (**a**); B,H,DL denote bifurcation sets, hysteresis sets, and double limit point sets, respectively.

**Figure 9:**
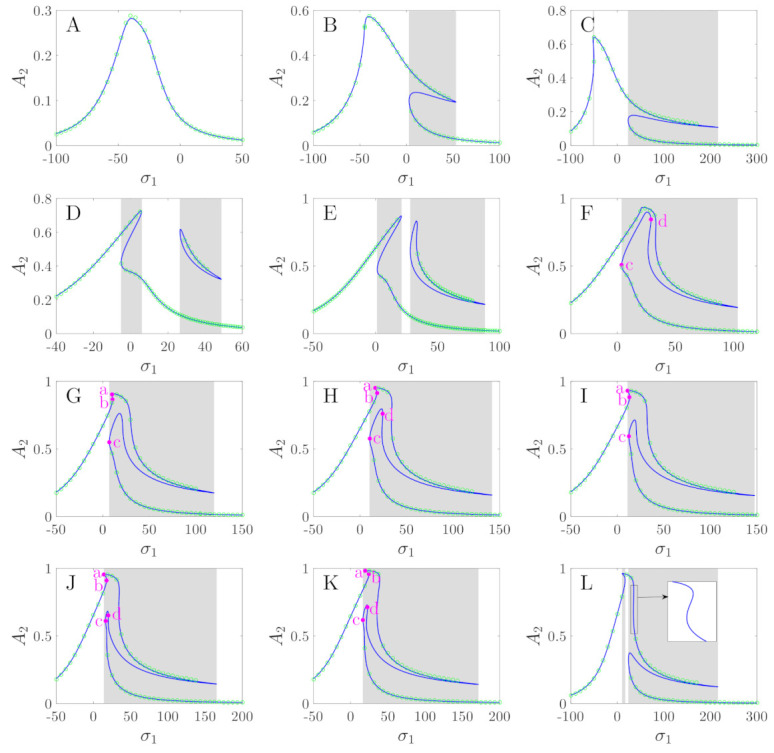
Bifurcation diagrams of the detecting amplitude variation with detuning parameters corresponding to different regions of Figure 8. The shaded areas denote the multi-solution areas, and the green circles denote the numerical solutions.

Figure 8 shows the transition sets on the driving–detecting voltage plane, which divide the plane into 12 persistent regions. Figure 9 presents the bifurcation diagrams corresponding to the persistent regions in Figure 8. The corresponding values of $E_{X}$ and $E_{Y}$ are given in Table 2. It can be seen that, corresponding to region A, there is only one solution of $A_{2}$ for different $\sigma_{1}$. However, in region B, corresponding to one interval of $\sigma_{1}$, the system may have three solutions. In the bifurcation diagram of region C, multiple solutions appear in two intervals of $\sigma_{1}$. In region D, a different solution branch appears, and in region E, folding happens in the added solution branch.

**Figure 10:**
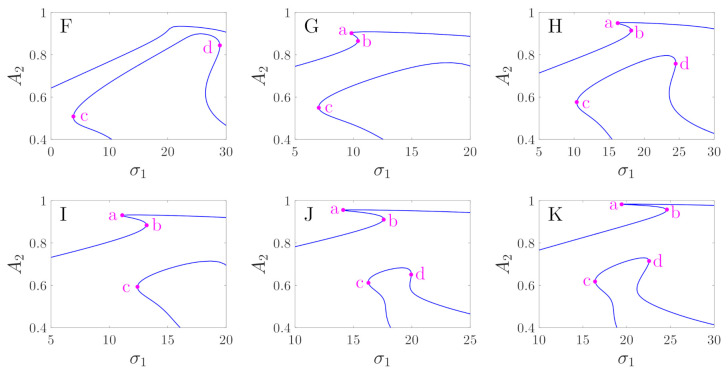
Local enlargements of subplots F through K of Figure 9.

In order to explain the differences in the bifurcation diagrams corresponding to regions F through K, we defined four key points as points a through d. They are all turning points of the bifurcation curves, as shown in Figure 9. Figure 10 shows the partial enlargements of the bifurcation curves. We can see in the bifurcation diagram of region F that points a and b do not exist. However, corresponding to regions G and I, point d does not exist. The differences in G and I are that point c in the curves of region G is on the left side of point a, and point c in the curves of region I is between a and b. Corresponding to regions H, J, and K, points a through d all exist, while in the curves of J, point c is between a and b, and the curves of regions H and K are to the left of point a. In region K, point d is between a and b, and point d of regions J and H is on the right of point b. The different positions of these turning points may cause different jumping phenomena as the driving frequency is increased or decreased. Figure 9 also gives the comparisons between the numerical solutions and the analytical solutions. It can be seen that the numerical solutions are in good agreement with the analytical solutions, except for the case in which the value of $\sigma_{1}$ is large. This is because in the calculation of Equation (32), we set Ω=1, but actually, $\Omega =1+\varepsilon \sigma_{1}$.

Micro-machined gyroscopes are not suitable for operation in multi-solution areas because the response may jump to other solutions, which may result in the gyroscope producing an incorrect output, as the system is disturbed. Figure 9 shows the multi-solution regions of the bifurcation curves, which are marked by the shaded areas. It can be seen in subplots D through L that most of the peak parts of the resonance curves are near multi-solution areas; thus, they do not meet the working requirements of the gyroscope. Only the bifurcation curves of regions A through C have large single-solution areas near the peak parts. Thus, in the following, we focus on the analysis of the response characteristics in regions A through C.

**Figure 11:**
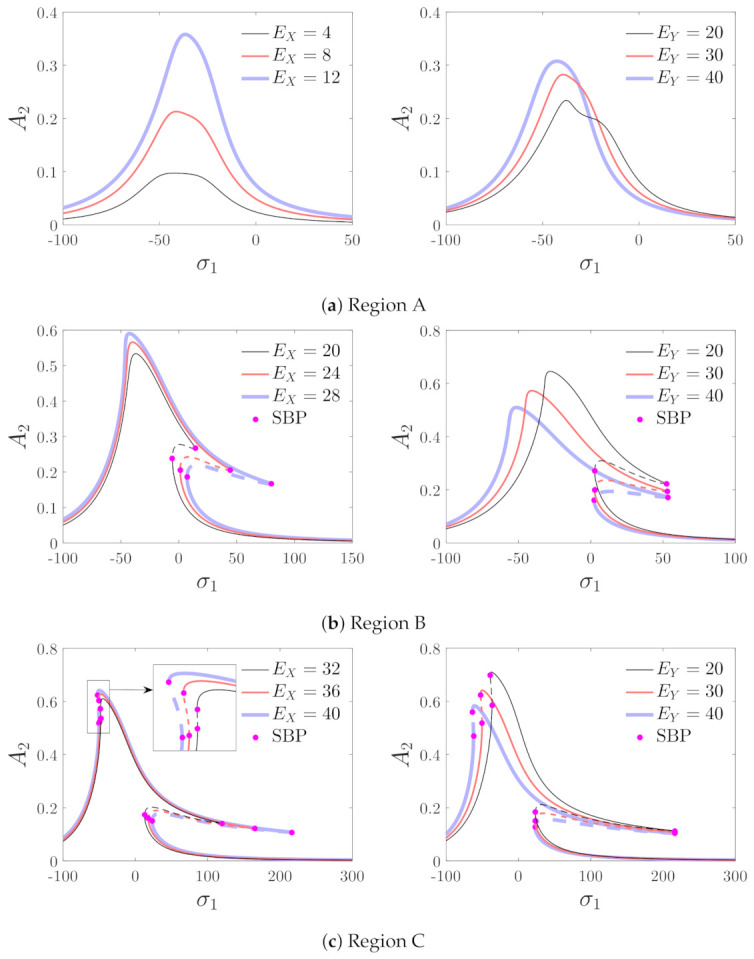
Influences of driving and detecting voltages on the amplitude–frequency curves; Region A: $E_{X}=10,E_{Y}=30$, Region B: $E_{X}=25,E_{Y}=30$, and Region C: $E_{X}=40,E_{Y}=30$.

The effects of the driving and detecting voltages on the response are analyzed in regions A through C. The results are presented in Figure 11. In region A, the peak amplitude of the curves increases with the driving and the detecting voltages. In the bifurcation diagrams of region B, two saddle–node bifurcation points appear on the right side of the curve, which indicates that jump phenomena occur with the variation in $\sigma_{1}$. The peak amplitude increases with the driving voltage and decreases with the detecting voltage. The effects of the driving and detecting voltages are opposite in regions A and B. Hence, the bifurcation analysis in this section is necessary. The peak frequency decreases with the driving and detecting voltages in region B. In region C, the saddle–node bifurcation points appear on both the left and right sides of the resonance curves, which means that jumping phenomena can take place two times—when the driving frequency is increased or decreased. The peak amplitude increases with the detecting voltage and decreases with the driving voltage.

**Figure 12:**
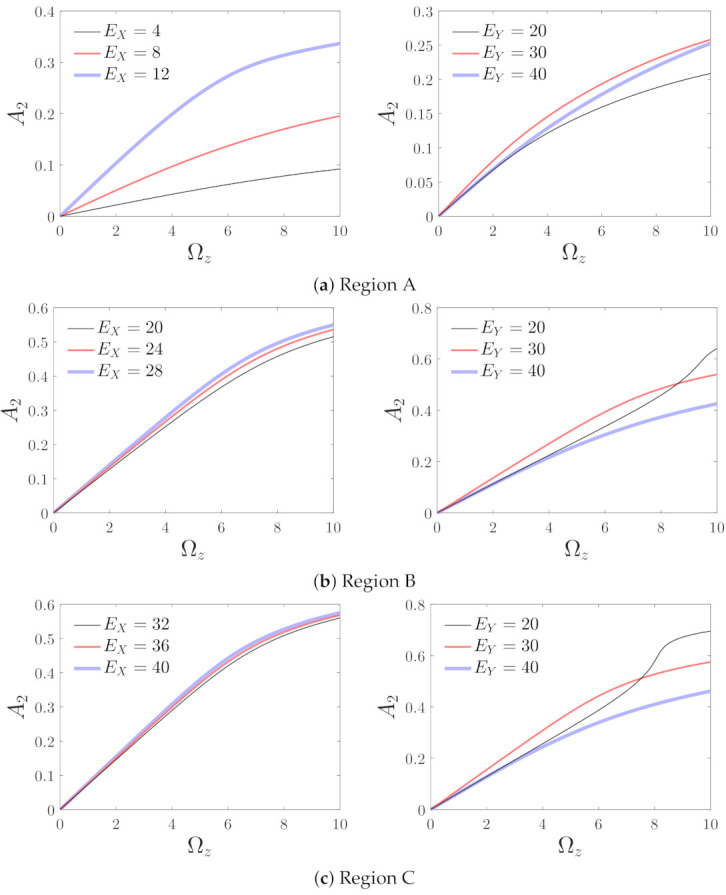
Variations in the detecting amplitude with the measured angular velocity corresponding to different parameter regions; Region A: $E_{X}=10,E_{Y}=30$, Region B: $E_{X}=25,E_{Y}=30$, and Region C: $E_{X}=40,E_{Y}=30$.

The variations in the detecting amplitude with the measured angular velocity are the most important for micro-machined gyroscopes. Figure 12 shows the influences of the non-dimensional angular velocity $\Omega_{z}$ on the detecting amplitude corresponding to different driving and detecting voltages. It can be observed in regions A through C, corresponding to any value of $\Omega_{z}$, that $A_{2}$ increases with the driving voltage. The effects of the detecting voltage are more complicated. In region A, and with a small value of $\Omega_{z}$ in regions B and C, a medium detecting voltage leads to a large amplitude, while the curve of $E_{Y}=20$ changes sharply with a large value of $\Omega_{z}$ in regions B and C. This change affects the linearity of the influence curve and is unfavorable for the operation of the gyroscope. Therefore, a small detecting voltage should be avoided in the B and C regions.

**Figure 13:**
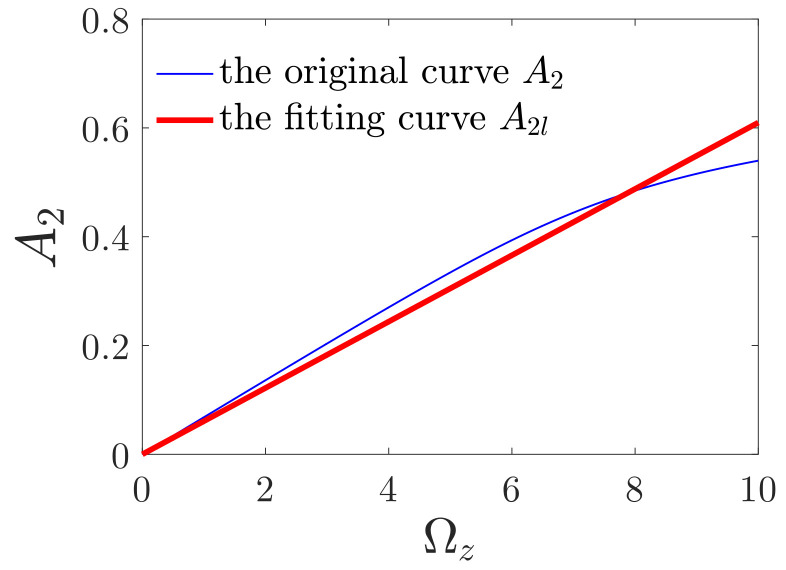
Least-square fitting of the influence curve of the angular velocity; $E_{X}=25,E_{Y}=30$.

In order to explain the influences of driving and detecting voltages on the response characteristics of the gyroscope clearly, mechanical sensitivity is considered. Mechanical sensitivity is the ratio of the detecting amplitude to the angular velocity [39,40]. However, for the nonlinear system, the ratio of the detecting amplitude to the angular velocity varies with the angular velocity. To calculate mechanical sensitivity, the least-square method is employed to fit the influence curves of the angular velocity linearly (shown in Figure 13). The fitting function is given as

(33) $$A_{2l}=S\Omega_{z},$$

where *S* is defined as mechanical sensitivity. Meanwhile, in order to illustrate the difference between the fitting line and the original curve, we defined the expression of nonlinearity of the influence curves, which is given as follows.

(34) $$\gamma =\frac{\max|A_{2}-A_{2l}|}{\max A_{2l}}$$

**Figure 14:**
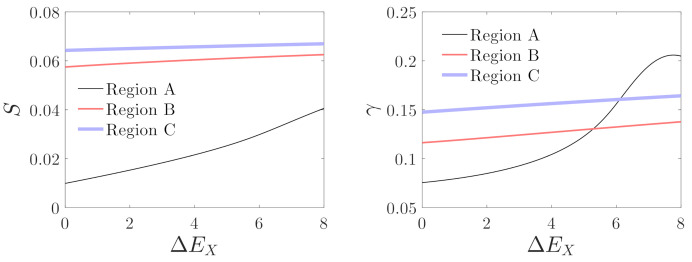
Influences of the driving voltage on mechanical sensitivity and nonlinearity; Region A: $E_{X}=\Delta E_{X}+4,E_{Y}=30$, Region B: $E_{X}=\Delta E_{X}+20,E_{Y}=30$, and Region C: $E_{X}=\Delta E_{X}+32,E_{Y}=30$.

**Figure 15:**
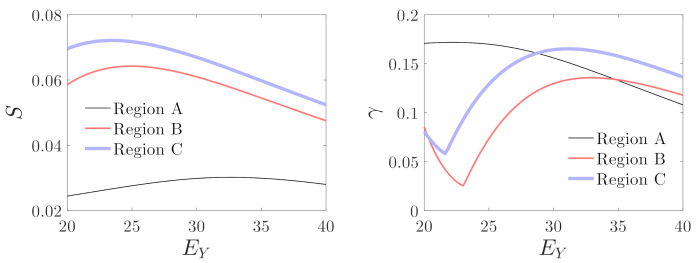
Influences of the detecting voltage on mechanical sensitivity and nonlinearity; Region A: $E_{X}=10$, Region B: $E_{X}=25$, and Region C: $E_{X}=40$.

Figure 14 and Figure 15 show the variations in mechanical sensitivity and nonlinearity with driving and detecting voltages in regions A, B, and C. It can be seen that mechanical sensitivity and nonlinearity increase with the driving voltage on the whole. However, mechanical sensitivity is much smaller for the operation of the micro-gyroscope in region A compared with the results in regions B and C. Nonlinearity decreases monotonously with the increase in detecting voltage in region A. However, the variations of nonlinearity are non-monotonous in regions B and C. With the increase in detecting voltage, mechanical sensitivity increases until it peaks, and thenceforth, it decreases. The nonlinearity decreases initially, followed by an increase, and then again decreases as the detecting voltage increases. Corresponding to $E_{Y}=20$ through 25 in regions B and C, the system may have the maximum mechanical sensitivity and minimum nonlinearity.

## 6. Conclusions

In this paper, the nonlinear dynamics of a micro-machined gyroscope system are presented with an approximate and numerical method that focuses on the effects of the driving and detecting voltages on the periodic motions and their bifurcations. Considering the nonlinear stiffness and the detecting electrostatic force, the dynamical equations of the micro-machined gyroscope are established with the Lagrange equation. The bifurcation of the equilibriums of the undisturbed system with the detecting voltage, as well as the pull-in effect, was studied, which showed that the system can have a stable periodic response only if the detecting voltage is kept within a certain range.

Through the method of averaging and the residue theorem, the dynamic equations with fractional terms were solved without Taylor expansion to find approximate analytical solutions. Numerical calculations verified the accuracy of the analytical solutions.

The influences of the driving and detecting voltages on the bifurcation characteristics of the system were studied through the singularity theory. Transition sets were given on the driving/detecting voltage plane, which was divided into 12 persistent regions. Analysis of the corresponding bifurcation diagrams showed that only three parameter regions met the operation requirements of the micro-machined gyroscope. In other regions that corresponded to a large driving voltage and small detecting voltage, most of the peak parts of the amplitude–frequency curves belonged to multi-solution areas.

The effects of the driving and detecting voltages on amplitude–frequency curves and the influence of these curves on angular velocity, as well as mechanical sensitivity and nonlinearity, were discussed for the three parameter regions. The results showed that in region A, which has a small driving voltage, mechanical sensitivity is much lower. In the other two regions, the variations in mechanical sensitivity and nonlinearity are analogous. It is possible that the system has a maximum mechanical sensitivity and minimum nonlinearity for an appropriate range of detecting voltages.

## Figures and Tables

**Table 1:** Expressions of the transition sets of the singularity theory.

Transition Sets	Expressions
Bifurcation sets B	$f=f_{A_{2}}=f_{\sigma_{1}}=0$
Hysteresis sets H	$f=f_{A_{2}}=f_{A_{2}A_{2}}=0$
Double limit point sets DL	$f\left(A_{2(i)},\sigma_{1}\right)=f_{A_{2(i)}}\left(A_{2(i)},\sigma_{1}\right)=0,i=1,2$ and $A_{2(1)}\neq A_{2(2)}$

**Table 2:** Values of $E_{X}$ and $E_{Y}$ corresponding to subplots A through L of Figure 9.

Subplots	$E_{X}$	$E_{Y}$	Subplots	$E_{X}$	$E_{Y}$
A	10	30	G	32	1.5
B	25	30	H	34	0.5
C	40	30	I	34.5	1
D	25	2	J	36	0.5
E	29	0.3	K	36.5	0.1
F	30.5	0.5	L	40	0.5

## Data Availability

The data that support the findings of this study are available from the corresponding author, [H. Zhang], upon reasonable request.

## Appendix A

$\begin{array}{c} M_{11}=-\frac{\xi_{X}}{2},\\ M_{12}=-\;\frac{G\Omega \;A_{20}\sin\left(\theta_{10}-\theta_{20}\right)+E_{X}\cos\theta_{1}}{2\Omega },\\ M_{13}=\frac{1}{2}\;G\cos\left(\theta_{10}-\theta_{20}\right),\\ M_{14}=\frac{1}{2}\;GA_{20}\sin\left(\theta_{10}-\theta_{20}\right),\\ M_{21}=\;\frac{2\;G\Omega \;A_{20}\sin\left(\theta_{10}-\theta_{20}\right)+3\;\beta_{X}{A_{10}}^{3}+2E_{X}\cos\theta_{1}}{4\Omega \;{A_{10}}^{2}},\\ M_{22}=-\frac{G\Omega \;A_{20}\cos\left(\theta_{10}-\theta_{20}\right)-E_{X}\sin\theta_{1}}{2\Omega \;A_{10}},\\ M_{23}=-\;\frac{G\sin(\theta_{10}-\theta_{20})}{2A_{10}},\\ M_{24}=\;\frac{GA_{20}\cos\left(\theta_{10}-\theta_{20}\right)}{2A_{10}},\\ M_{31}=-\frac{1}{2}\;G\cos\left(\theta_{10}-\theta_{20}\right),\\ M_{32}=\frac{1}{2}\;A_{10}G\sin\left(\theta_{10}-\theta_{20}\right),\\ M_{33}=-\frac{\xi_{Y}}{2},\\ M_{34}=-\frac{1}{2}A_{1}G\sin\left(\theta_{10}-\theta_{20}\right),\\ M_{41}=-\frac{G\sin(\theta_{10}-\theta_{20})}{2A_{20}},\\ M_{42}=-\;\frac{A_{10}G\cos\left(\theta_{10}-\theta_{20}\right)}{2A_{2}},\\ M_{44}=\;\frac{A_{10}G\cos\left(\theta_{10}-\theta_{20}\right)}{2A_{2}}\\ M_{43}=\;\frac{\left[2\;G\Omega \;A_{10}\sin\left(\theta_{10}-\theta_{20}\right)+3\;\beta_{Y}{A_{20}}^{3}\right]{\left(1-{A_{20}}^{2}\right)}^{5/2}-6\;{A_{20}}^{3}E_{Y}}{4{A_{20}}^{2}{\left(1-{A_{20}}^{2}\right)}^{5/2}\Omega }. \end{array}$
